# Primary hyperaldosteronism induces congruent alterations of sodium homeostasis in different skeletal muscles: a ^23^Na-MRI study

**DOI:** 10.1530/EJE-22-0074

**Published:** 2022-03-07

**Authors:** Martin Christa, Stefanie Hahner, Herbert Köstler, Wolfgang Rudolf Bauer, Stefan Störk, Andreas Max Weng

**Affiliations:** 1 University and University Hospital Würzburg, Comprehensive Heart Failure Center, Würzburg, Germany; 2 University Hospital Würzburg, Medical Clinic and Polyclinic for Internal Medicine I, Würzburg, Germany; 3 Department of Diagnostic and Interventional Radiology, University Hospital Würzburg, Würzburg, Germany

## Abstract

**Background:**

Sodium homeostasis is disrupted in many cardiovascular diseases, which makes non-invasive sodium storage assessment desirable. In this regard, sodium MRI has shown its potential to reveal differences in sodium content between healthy and diseased tissues as well as treatment-related changes of sodium content. When different tissues are affected disparately, simultaneous assessment of these compartments is expected to provide better information about sodium distribution, reduce examination time, and improve clinical efficiency.

**Objectives:**

The objectives were (1) to investigate sodium storage levels in calf and pectoral muscle in healthy controls and patients and quantify changes following medical treatment and (2) to demonstrate homogeneous disruption in skeletal muscle sodium storage in patients with primary hyperaldosteronism (PHA).

**Methods:**

We assessed sodium storage levels (relative sodium signal intensity, rSSI) in the calf and pectoral muscles of eight patients with PHA prior and after treatment and 12 age- and sex-matched healthy volunteers.

**Results:**

Calf and pectoral muscle compartments exhibited similar sodium content both in healthy subjects (calf vs pectoral rSSI: 0.14 ± 0.01 vs 0.14 ± 0.03) and PHA patients (calf vs pectoral rSSI: 0.19 ± 0.03 vs 0.18 ± 0.03). Further, we observed similar treatment-related changes in pectoral and calf muscles in the patients (proportional rSSI change calf: 26%; pectoral: 28%).

**Conclusion:**

We found that sodium was distributed uniformly and behaved equally in different skeletal muscles in Conn’s syndrome. This allows to measure both heart and skeletal muscle sodium signals simultaneously by a single measurement without repositioning the patient. This increases ^23^Na-MRI’s clinical feasibility as an innovative technique to monitor sodium storage.

## Introduction

Many cardiovascular diseases are associated with imbalances in sodium homeostasis, thus raising the clinical need to non-invasively assess the sodium content not only within the heart but also in other organs. Sodium MRI (^23^Na-MRI) appears to be promising in this regard as recently suggested by our group based on investigations in patients with primary hyperaldosteronism (PHA) (1). PHA is not only characterized by high salt intake and hypertension but also by an elevated risk for cardiovascular events (2). A recent study highlighted the particular role of sodium by demonstrating lower rates of cardiovascular events, stroke, or death among persons with reduced sodium intake (3). Recently, our group reported an increased tissue sodium content (TSC) in the skin, the skeletal muscle, and the myocardium of PHA patients compared to healthy controls. Moreover, we had found that the sodium concentration of different body tissues was disparately affected (1).

To enhance understanding of sodium metabolism, improve risk stratification in patients, and study the pathophysiological alterations introduced by therapeutic strategies (e.g. mineralocorticoid receptor antagonists, sodium-glucose linked transporter 2 [SGLT-2] inhibitors, diuretics), the exact quantification of sodium content in different tissues besides the heart is required. Important tissues for sodium storage are myocardium, skeletal muscle, and skin, with the latter two tissues serving in particular as a reference for the first. Skeletal muscle and skin are frequently assessed from the calf region (1, 4, 5). However, when the heart is in focus, this implies a considerable prolonged imaging time, due to repositioning of the patient. Hence, simultaneous assessment of multiple tissues could greatly advance the practicability of ^23^Na-MRI. This would apply to cardiovascular diseases including hypertension, PHA, and heart failure, where a homogeneous disruption of sodium homeostasis is assumed.

A promising candidate for simultaneous assessment is the pectoral muscle, due to its vicinity to the myocardium. In this report, we show that the assessment of sodium content in the pectoral muscle by ^23^Na-MRI is feasible and that its sodium content is equivalent to the ‘reference standard’ calf muscle in healthy controls and patients. The congruent disruption of sodium homeostasis across skeletal muscle compartments supports to utilize the pectoral muscle as an appropriate reference for multi-site quantification of sodium storage.

## Methods

### Subjects and study design

The present analysis is based on patients recruited for the recently published MYSTIC study (1). The study is registered at the German Clinical Trial Register (DRKS00010946). The methodological details including MRI setup and sequence parameters have been described in detail (1). The Ethics Committee of the University Hospital Würzburg approved the study (#220/13). Informed consent was obtained from each patient/healthy volunteer after full explanation of the purpose and nature of all procedures used in this study. In brief, the study was conducted with eight patients with PHA (Conn’s syndrome) and 12 healthy controls (HC). All subjects underwent ^23^Na-MRI of the calf and thorax to detect and quantify tissue sodium signals of the calf and pectoral muscle. PHA patients were treated with either unilateral adrenalectomy or drug treatment (mineralocorticoid receptor antagonists). Six out of the eight PHA patients underwent follow-up examination after at least 4 months of treatment.

### ^23^Na- and ^1^H-magnetic resonance imaging

^23^Na-MRI of the calf and thorax was performed on a clinical 3T whole body scanner (Magnetom PRISMA, Siemens) using a dual tuned ^23^Na/^1^H surface coil (Rapid Biomed, Rimpar, Germany). For thoracic MRI, patients were examined in prone position to minimize breathing motion with a 3D gradient echo sequence for ^23^Na. The same sequence was used for the ^23^Na-MRI of the calf muscle. A vial with 100 mmol/L of sodium as reference for quantification was scanned with every participant. Tissue sodium is presented as the ratio:


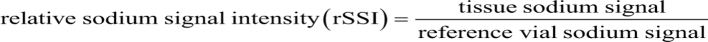


**Figure 1:**
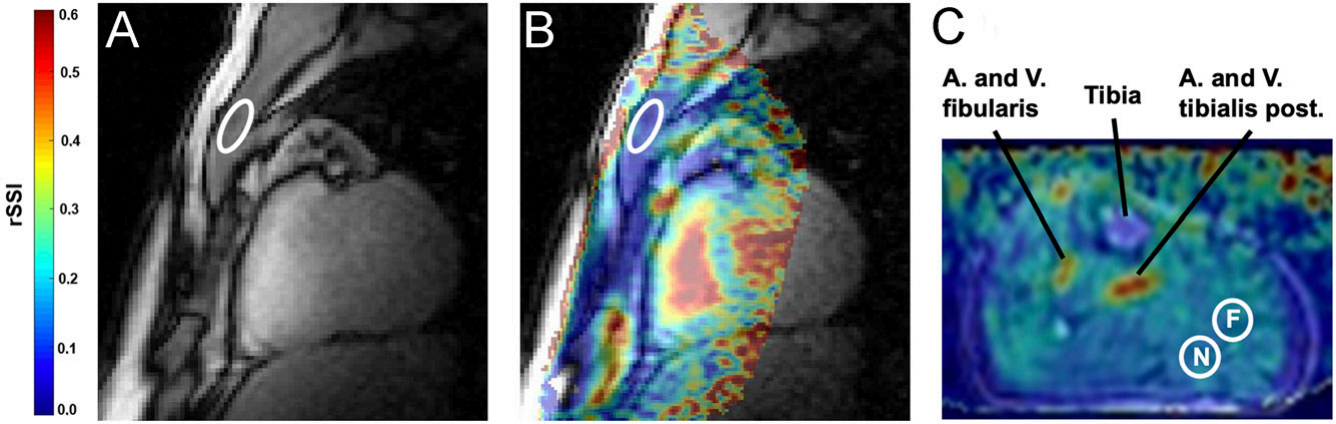
Exemplary ROI placement in the pectoral muscle (A and B) and in the calf (C). The colored overlay in (B) visualizes the parts of the FOV that were used for sodium quantification. In the calf, two ROIs (ROI near (N), ROI far (F)) were averaged to determine rSSI values (C). FOV, field of view; ROI, region of interest; rSSI, relative sodium signal intensity.

For each sodium image, a corresponding proton image was co-registered to allow an accurate placement of regions of interest (ROIs). In the calf, two ROIs were placed that contained no skin, fat, blood, or bone but only one of the muscles. The mean signal value from both ROIs was used for further evaluation. As shown earlier (1), the receive coil sensitivities were corrected and no resulting difference in sodium signal intensity was observed between different ROIs with different distances to the receive coil. Similarly, in the pectoral muscle, ROIs were also placed avoiding skin, fat, blood, bone, and cartilage. Therefore, no restriction in size (neither minimum nor maximum) was imposed to be able to place a meaningful ROI. Figure 1 shows an exemplary ROI placement in the pectoral and the calf muscle.

### Statistical analysis

To compare the rSSIs of pectoral and calf muscle in groups of PHA and HC, the Mann–Whitney *U* test was used. To compare calf and pectoral muscle sodium levels prior and after treatment (PHA group at baseline (BL) and follow-up (FUP)), a paired Wilcoxon signed-rank test was applied. To assess the intra-individual agreement of the two muscle compartments, we used a commonly recommended approach: First, Spearman’s rho correlation coefficient was calculated, assuming nonparametric distribution. Second, we calculated the mean of the differences according to Bland and Altman. All statistical analyses were carried out using SPSS 26 (IBM Corp.).

## Results

**Figure 2:**
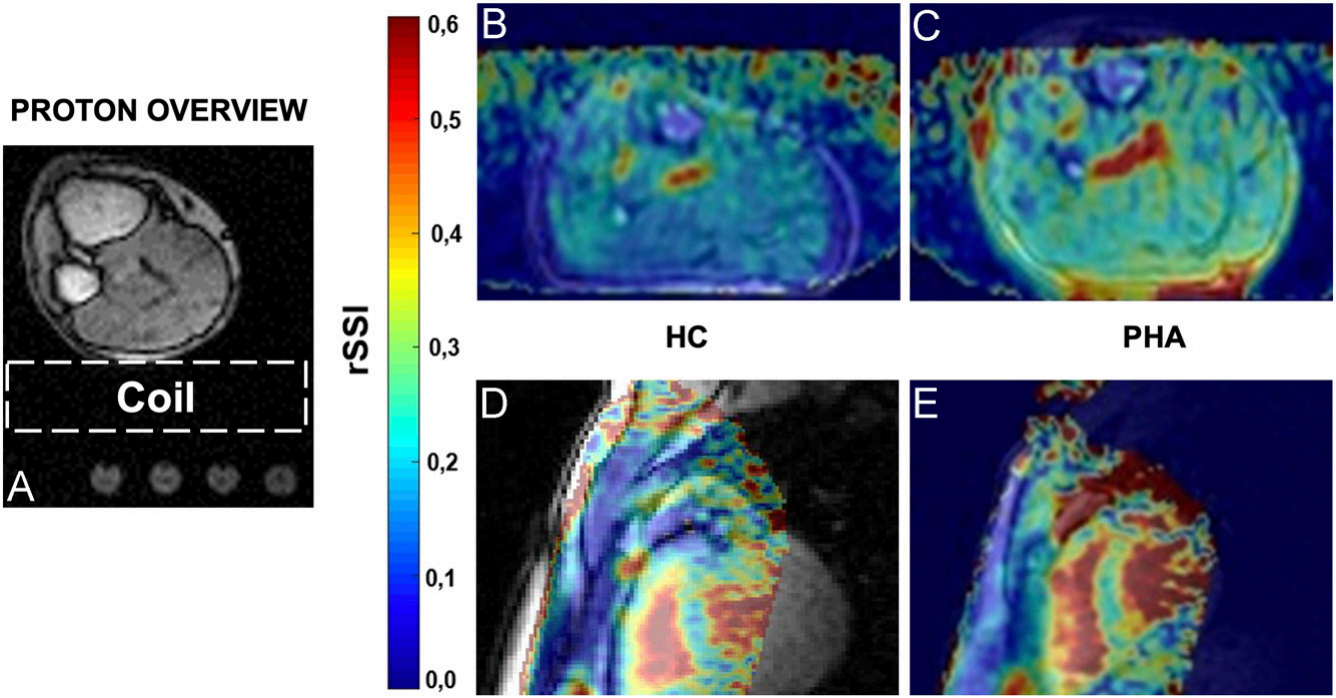
The experimental setup for the calf as well as the reference vials below the coil are shown (A). The vial on the left contained 100 mmol/L of NaCl and was used to calculate the rSSI maps. The same setup was used for the chest, with the patients in prone position. (B) and (C) provide a comparison of the calf muscles of a HC and a PHA patient prior treatment, whereas (D) and (E) demonstrate exemplary images of the pectoral muscle and the heart, correspondingly. HC, healthy controls; PHA, primary hyperaldosteronism; rSSI, relative sodium signal intensity.

Both groups, PHA and controls, did not significantly differ regarding age and sex. The mean (±s.d.) age of healthy volunteers was 57.6 ± 9.9 years and was 54.3 ± 7.0 years among PHA patients. The rSSIs of the calf and the pectoral muscle in PHA patients were similar at BL (calf: 0.19 ± 0.03, pectoral: 0.18 ± 0.03) and again similar at the FUP assessment (calf: 0.14 ± 0.02; pectoral: 0.13 ± 0.02). Thus, the pectoral muscle in PHA patients demonstrated significantly increased rSSI compared to HC (PHA vs HC pectoral rSSI: 0.18 ± 0.03 vs 0.14 ± 0.03; *P*  = 0.016) at BL, as already observed for the calf muscles (PHA vs HC calf rSSI: 0.19 ± 0.03 vs 0.14 ± 0.01; *P*  = 0.001). Furthermore, the pectoral rSSI significantly declined when comparing PHA BL measurements with FUP values (PHA BL vs FUP pectoral rSSI: 0.18 ± 0.03 vs 0.13 ± 0.02; *P*  = 0.028). FUP rSSI levels in PHA pectoral muscle were comparable to pectoral rSSI levels in HC. Again, similar behavior had been reported earlier for the calf muscle: PHA BL vs PHA FUP calf rSSI: 0.19 ± 0.03 vs 0.14 ± 0.02; *P*  = 0.046 (1). Figure 2 provides a scheme of the experimental setup and demonstrates the difference in rSSI between HC and PHA.

**Figure 3:**
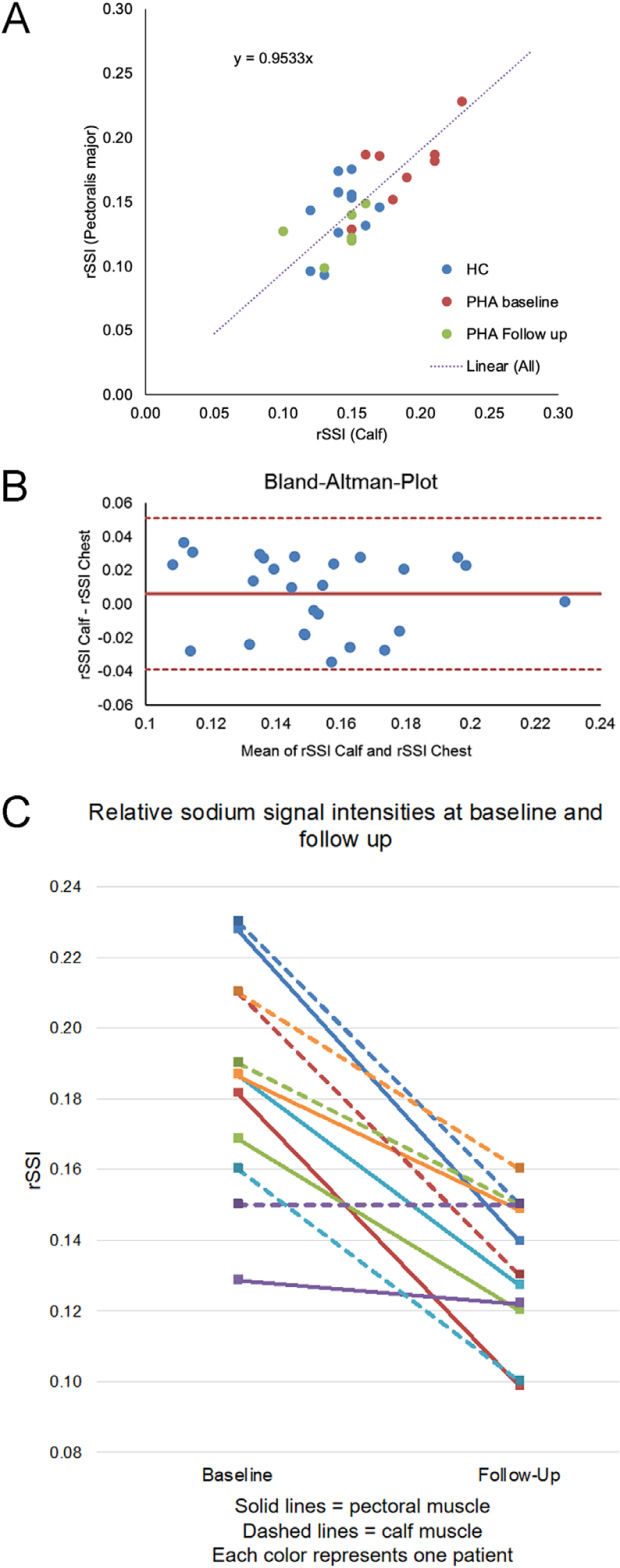
(A) rSSI values of the pectoral and calf muscle for each individual are plotted (Spearman’s rho 0.63; *P*  = 0.001). Of note, two HC had the same pair of values, thus only 11 HC are visible. (B) Bland–Altman plot of rSSI measurements comparing calf and pectoralis major showing excellent agreement. (C) Intra-individual changes in pectoralis major and calf rSSIs following PHA-directed treatment. Each patient is represented by one color. Solid line represents the pectoral muscle, the corresponding dashed line the calf muscle of a respective patient. HC, healthy controls; PHA, primary hyperaldosteronism; rSSI, relative sodium signal intensity.

In Fig. 3A, the skeletal muscle sodium signals of calf and pectoral muscle of all examinations (*n*  = 26) were plotted, and a strong correlation for rSSI values in both compartments became evident: Spearman’s rho 0.63, *P*  < 0.001. We calculated the mean of the differences and plotted them against the difference of both measured rSSI values (Fig. 3B). Bland–Altman analysis showed favorable concordance between both muscle compartments, with a mean of differences of 0.01 (95% CI: –0.04, 0.05).

Finally, we compared pectoral and calf muscle rSSIs prior and after treatment in PHA patients. Both muscle compartments exhibited similar intra-individual treatment-related changes, as plotted in Fig. 3C. Decreases in calf rSSI and the pectoralis major muscle rSSI following PHA-directed treatment (*n* = 6) yielded a strong correlation: rho 0.62, *P*  = 0.004. The mean relative rSSI change of the calf was −26%, and −28% in the pectoral muscle.

## Discussion

We assessed the pectoral major muscle’s sodium storage and compared it to the calf. To the best of our knowledge, this is the first study comparing sodium storage of skeletal muscle tissue in different parts of the body and thereby reporting values for sodium storage in the healthy pectoral muscle. As the calf is easily accessible for ^23^Na-MRI investigation, nearly all of the yet reported values of muscle sodium content were derived from the lower extremities. Therefore, no values for the pectoral muscle were available up to now.

In PHA, an increased sodium storage was demonstrated in the calf (1). Our findings now show similarly increased rSSI values in the pectoral muscle in PHA patients compared to healthy volunteers. Furthermore, the rSSI of the pectoral muscle in PHA patients declined after treatment to levels comparable to HC. These changes in the pectoral rSSI paralleled the changes in the calf.

To our knowledge, there is only one study that reported on the variability of sodium storage in the calf of healthy subjects. The authors explained the observed variance by differences in the intracellular sodium or heterogeneity in intracellular volume fractions due to different patterns of muscle fiber composition (6). In contrast, when comparing muscles from two different body parts (lower extremities and body core), we not only failed to see any significant differences between calf and pectoral muscle but rather observed the same behavior regarding PHA and the related treatment.

As described by previous studies (4, 7), there are age- and sex-related differences in skeletal muscle sodium storage. We accounted for this by an age- and sex-matched recruiting strategy of HC and PHA patients. For the reported intra-individual comparison of calf and pectoralis muscle, one may assume that both compartments are affected similarly by the co-variables age and sex, which therefore do not necessarily need to be taken into account. Furthermore, recruiting PHA patients who underwent standardized diagnostic workup and treatment (for details see Ref. (2)) ensured a well-phenotyped patient sample. Thus, despite the small sample size, post hoc power calculations resulted in a power of 0.98 for a difference in calf rSSI of HC against PHA and 0.96 for the difference in pectoral rSSI.

Our results show for the first time that sodium storage levels in the pectoral muscle of healthy volunteers and PHA patients are congruent with sodium levels in their respective calf muscle. Additionally, we could demonstrate that the pectoral and calf rSSI are equally affected by PHA-directed treatment. This suggests a homogeneous disruption of the skeletal muscle sodium storage, in the case of Conn’s syndrome.

### Limitations

As sodium is a nucleus with a bi-exponential signal decay (T2 relaxation) (8), for ^23^Na sequences at least an echo time (TE) of 0.4 ms or shorter is needed to allow total quantification. The sequence used for this study had a TE of 2.01 ms and thus may not have captured the complete ^23^Na signal. To account for this, we intentionally did not report absolute TSC in mmol per volume but rather the relative signal strength compared to the 100 mmol/l reference vial. As the relative sodium signal strength might suffer from a low signal-to-noise ratio (SNR) or local signal variations, the whole setup was validated in a previous study in terms of repeatability and accuracy (1).

As we focused on a disease which assumedly affects the body’s skeletal sodium storage in a global manner, our results do not apply for diseases with definite inhomogeneous affection of the skeletal muscle system, and, hence, sodium homeostasis, as demonstrated, for example, in neuromuscular diseases affecting singular muscles or compartments (5, 9). Examinations of the thoracic organs (e.g. lung or heart) usually benefit from prospective or retrospective gating or some method of post-processing that is applied to compensate for motion (artifacts) (10). As our patients were examined in prone position, movement of the pectoral muscle was minimized. For future studies, especially when examining the individual in supine position, the protocol could be adapted to evaluate the effects/benefits of motion correction on the pectoral muscle sodium signal. Lastly, tissue sodium assessment was performed under resting conditions. Bansal *et al*. demonstrated an increased ^23^Na signal in skeletal muscle after exercise (11). Thus, our findings may only apply to skeletal muscle at rest.

## Conclusion/perspectives

We compared the calf and pectoral muscles regarding (a) their sodium storage pattern and (b) their ability to mirror treatment-related changes in tissue sodium content at rest. We could show similar BL rSSIs of tissue sodium content between healthy calf and pectoral muscles, and congruently increased rSSI levels of both muscle compartments in patients suffering from PHA. Furthermore, tissue sodium signal declined concurrently in both muscles after PHA0- directed treatment. Our findings allow utilizing the pectoral muscle as reference for skeletal muscle sodium content. This facilitates the simultaneous assessment of myocardial and skeletal muscle tissue, which significantly decreases examination time in multi-tissue sodium MRI investigations, quantification of treatment effects, or monitoring of disease progression.

## Declaration of interest

A M W and H K received a grant from Siemens Healthcare outside the submitted work. M C received a speaker’s grant from Pfizer outside the submitted work. All other authors declare no conflict of interest.

## Funding

This study was supported by grants of the Comprehensive Heart Failure Center (CHFC) Würzburg to M C and H K. The CHFC was supported by the Federal Ministry of Education and Research (grants #01EO1004, #01EO1504). M C was supported by the German Research Council (Deutsche Forschungsgemeinschaft, DFG) supported Clinician Scientist Program UNION CVD (#413657723).

## Data availability

The data underlying this article will be shared on reasonable request to the corresponding author.
